# Features of the Skin Microbiota in Common Inflammatory Skin Diseases

**DOI:** 10.3390/life11090962

**Published:** 2021-09-14

**Authors:** Iva Ferček, Liborija Lugović-Mihić, Arjana Tambić-Andrašević, Diana Ćesić, Ana Gverić Grginić, Iva Bešlić, Marinka Mravak-Stipetić, Iva Mihatov-Štefanović, Ana-Marija Buntić, Rok Čivljak

**Affiliations:** 1 Department of Dermatovenereology, Sestre Milosrdnice University Hospital Center, 10000 Zagreb, Croatia; liborija@sfzg.hr (L.L.-M.); diana.vrancic@gmail.com (D.Ć.); ivaaabukvic@gmail.com (I.B.); 2 Department of Ophthalmology, Sestre Milosrdnice University Hospital Center, 10000 Zagreb, Croatia; 3 School of Dental Medicine, University of Zagreb, 10000 Zagreb, Croatia; arjana.tambic@bfm.hr (A.T.-A.); mravak@sfzg.hr (M.M.-S.); iva.mihatov@kbcsm.hr (I.M.-Š.); 4 Department of Clinical Microbiology, University Hospital for Infectious Diseases “Dr. Fran Mihaljević”, 10000 Zagreb, Croatia; 5 Department of Clinical Microbiology, Sestre Milosrdnice University Hospital Center, 10000 Zagreb, Croatia; ana.gveric.grginic@kbcsm.hr; 6 Department of Pediatrics, Sestre Milosrdnice University Hospital Center, 10000 Zagreb, Croatia; ana.marija.buntic@gmail.com; 7 Department for Respiratory Infections, University Hospital for Infectious Diseases “Dr. Fran Mihaljević”, 10000 Zagreb, Croatia; rok.civljak@bfm.hr; 8 Department for Infectious Diseases, University of Zagreb School of Medicine, 10000 Zagreb, Croatia

**Keywords:** skin microbiota, skin diseases, facial skin, inflammatory skin diseases, atopic dermatitis, seborrheic dermatitis, rosacea, acne vulgaris, perioral dermatitis, periorificial dermatitis, periocular dermatitis, psoriasis

## Abstract

Many relatively common chronic inflammatory skin diseases manifest on the face (seborrheic dermatitis, rosacea, acne, perioral/periorificial dermatitis, periocular dermatitis, etc.), thereby significantly impairing patient appearance and quality of life. Given the yet unexplained pathogenesis and numerous factors involved, these diseases often present therapeutic challenges. The term “microbiome” comprises the totality of microorganisms (microbiota), their genomes, and environmental factors in a particular environment. Changes in human skin microbiota composition and/or functionality are believed to trigger immune dysregulation, and consequently an inflammatory response, thereby playing a potentially significant role in the clinical manifestations and treatment of these diseases. Although cultivation methods have traditionally been used in studies of bacterial microbiome species, a large number of bacterial strains cannot be grown in the laboratory. Since standard culture-dependent methods detect fewer than 1% of all bacterial species, a metagenomic approach could be used to detect bacteria that cannot be cultivated. The skin microbiome exhibits spatial distribution associated with the microenvironment (sebaceous, moist, and dry areas). However, although disturbance of the skin microbiome can lead to a number of pathological conditions and diseases, it is still not clear whether skin diseases result from change in the microbiome or cause such a change. Thus far, the skin microbiome has been studied in atopic dermatitis, seborrheic dermatitis, psoriasis, acne, and rosacea. Studies on the possible association between changes in the microbiome and their association with skin diseases have improved the understanding of disease development, diagnostics, and therapeutics. The identification of the bacterial markers associated with particular inflammatory skin diseases would significantly accelerate the diagnostics and reduce treatment costs. Microbiota research and determination could facilitate the identification of potential causes of skin diseases that cannot be detected by simpler methods, thereby contributing to the design and development of more effective therapies.

## 1. Introduction

Many relatively common chronic inflammatory skin diseases manifest on the face, impairing patient appearance and quality of life. Given the yet unexplained pathogenesis and numerous factors involved, these diseases often present therapeutic challenges. There has recently been extensive discussion on the skin microbiome as an important factor in disease development. The term “microbiome” comprises the totality of microorganisms (microbiota), their genomes, and environmental factors in a particular environment [1]. "Human microbiota", on the other hand, refers to the sum of all the microorganisms living on/in our body and is a source of genetic diversity, a modulator of health and disease, a fundamental component of immunity and an entity that affects metabolism and modulates drug interactions. A large number of microorganisms (bacteria, fungi, viruses) are found on the surface of or inside numerous human tissues and fluids, including skin, mammary glands, placenta, semen, uterus, ovarian follicles, lungs, saliva, oral mucosa, conjunctiva, and the biliary and gastrointestinal tracts. The largest share [2] of the microbiota is made up of bacteria, followed by fungi, viruses, and arthropods. Changes in human skin microbiota composition and/or functionality are believed to trigger immune dysregulation, and consequently an inflammatory response, thereby playing a potentially significant role in the clinical manifestations of inflammatory skin diseases [3]. Knowing the characteristics of the skin microbiome could therefore be crucial for understanding the occurrence and treatment of these skin diseases, making this an appropriate subject for research. The purpose of this review is to present the current knowledge on the characteristics of the skin microbiome in inflammatory skin diseases, taking into consideration that therapeutic effects on microbiome imbalance could contribute to disease improvement and sanitation.

## 2. Microbiota Analysis Methods

In gathering information about the microbiome, following human genome sequencing, an important step was to determine its variations and link them to diseases, with emphasis on the analysis of the microbiome’s contribution to human health and disease [4]. Research on the human microbiome is carried out in order to identify the microorganisms that inhabit certain regions of the human body. Current knowledge of the psoriasis and atopic dermatitis-associated microbial community has been obtained until recently by conventional culture-dependent studies, suggesting an association of several microorganisms with disease exacerbation, including *Staphylococcus aureus, Streptococcus pyogenes*, and fungi, such as *Malassezia* [5,6]. Cultivation methods have traditionally been used in studies of bacterial microbiome species, but a large number of bacterial strains cannot be grown in the laboratory. The culture-dependent methods performed under standard laboratory conditions can detect fewer than 1% of bacterial species [7]. The difficulties of cultivation lie in the slow and demanding growth of some bacteria, i.e., the dependence of growth on other species and the great influence of demanding cultivation conditions [8]. Consequently, the actual presence of bacterial species within the microbiome is often underestimated. A significant contribution to microbiome research has been made by developments in molecular biology and bioinformatics, as well as the reduction of sequencing costs. Thanks to the metagenomic approach, today, we are able to detect bacteria that cannot be cultivated. The term metagenomics refers to the use of modern genomic techniques for sequencing large sets of genes in a sample, without the need to isolate a particular species [9].

The methods for identifying microorganisms include quantitative polymerase chain reaction (PCR), denaturing gradient gel electrophoresis (DGGE), terminal restriction fragment length polymorphism (T-RFLP) and sequencing. The most commonly used molecular method independent of cultivation today is 16S rRNA gene sequencing, which is based on the knowledge that the DNA sequence varies among conserved 16S rRNA regions, depending on the bacterial species. The emphasis is placed on the importance of selecting the hypervariable region of 16s rRNA for sequencing. While the V1–V3 regions are more suitable for studying the skin microbiome, the V4 region is more useful for studying the intestinal microbiome [10,11]. The two approaches to 16S rRNA sequencing are targeted sequencing, i.e., next generation sequencing and the shotgun method. Third-generation methods offer the possibility of single-molecule real time sequencing of longer reads. The most significant platforms of that type are PacBio RS II (Pacific Biosciences) and MinION nano-pore devices (Oxford Nanopore Technologies), which do not require DNA amplification by PCR [12]. In addition to metagenomic methods, other meta-omic methods are used in the study of the taxonomic structure, and especially the functional capacity of the microbiome, namely metatranscriptomics, metaproteomics, and metabolomics. These modern molecular and bioinformatics technologies including the whole genome shotgun metagenomic sequencing (WGS metagenomic sequencing) of bacterial communities will define microorganisms’ genetic diversity and their relationship between commensal symbiotic and pathogenic microbiome. In addition to microbial composition, structure, and functionalities, novel technologies are providing significant insight into immune system functions and homeostasis alterations.

In determining the potential impact of the microbiome at the onset of a disease, it is important to become acquainted with the microbiome when a patient is healthy. Microbiome research is an area of interest of many branches of medicine. Large-scale research projects, such as the human microbiome project, have provided insight into the specificity and importance of the microbiome and laid foundation for future research and new knowledge [13]. This contributes to further clinical diagnostic and therapeutic applications of the acquired knowledge, since a number of diseases (types I and II diabetes, obesity, rheumatoid arthritis, inflammatory bowel disease, depression) are associated with dysbiosis, i.e., changes in the stability and the content of the intestinal, skin, and oral microbiomes. [14,15,16,17,18,19]. Research results of microbiome’s structural and functional effects on the skin and disease development provide basis for future treatment strategies, e.g., fecal microbiota transplantation as a successful application of microbiome knowledge for therapeutic purposes [20]. It could also be used in the treatment of skin diseases.

## 3. Current Knowledge of the Characteristics of the Skin Microbiome

Our knowledge of the skin microbiome is closely related to our knowledge of the skin structure and its layers: the epidermis, dermis, and the deeper subcutaneous tissue of the hypodermis, which together form physical and chemical barriers to external pathogens [21]. There is also an immune barrier that encompasses the temporary nonspecific component (innate immune response) and the highly specific long-acting component (adaptive immune response, also known as acquired immunity). The innate immune system, as the first line of defense, is designed to directly and rapidly respond to foreign pathogens by activating recognition systems and effector mechanisms. A unique feature of the adaptive immune response, which develops more slowly, is its ability to generate and retain memory. Therefore, it mounts a stronger antigen-specific response when the innate immune response fails to eliminate pathogens. [22]. When in symbiosis with its host, the human skin microbiota acts to preserve skin barrier functions. However, once this barrier is breached by intrinsic or extrinsic factors, it is capable of promoting both the innate and the adaptive immune response to maintain homeostasis. In addition to controlling the release of some antimicrobial peptides, skin-resident microbes are also capable of regulating components of the complement system and exacerbating skin inflammation via the recruitment of neutrophils and production of interleukins. [23]. The skin microbiome, i.e., the bacteria, fungi, viruses, and arthropods that colonize human skin, evidently has an important role. All these microorganisms play a part in modulating the immune response and, according to recent research, can be found not only in the epidermis, but also in deeper layers of the skin, dermis, and subcutaneous tissue. That means that they cross the skin barrier, interact with cells of the deeper layers, and affect their homeostasis [24]. The specificity of the microbiome of the epidermis in relation to the microbiome of the dermis is also noted. While the microbiome of the epidermis is strongly influenced by environmental factors, the microbiome of the dermis is more stable and less susceptible to change. Initial research suggests that the microbiome of the dermis is of uniform composition, regardless of body localization [25].

The very birth of a child can have an impact on the characteristics of the microbiome. For example, the mode of delivery can determine the composition of the skin microbiome of the newborn child. Immediately after birth, the skin of the newborn is exposed to environmental microorganisms that begin to inhabit/colonize it, creating a host-microorganism homeostasis. The microbiome of the skin of newborns born vaginally is similar to the vaginal microbiome of the mother, whereas the microbiome of children delivered by caesarean section shows similarities to the skin microbiome of the mother [26]. The skin microbiome of premature infants has its own specifics. It is richest in bacteria of the phyla *Firmicutes* (genus *Staphylococcus*) and *Bacteroidetes* (genus *Flavobacterium*). Compared to the skin of term infants, it contains a larger share of bacteria belonging to the *Firmicutes* phylum. Those of the *Proteobacteria* phylum are relatively sparse. Furthermore, the skin of preterm infants (samples collected from the forehead area, cubital fossa and gluteal region) has relatively copious bacteria of the *Staphylococcus*, *Corynebacterium*, and *Prevotella* genera, and sparse *Brevundimonas*, *Flavobacterium,* and *Sphingobacterium* species, compared to term-newborns’ skin [27]. Research has demonstrated that, the predominant phylum found on the skin of healthy infants is the *Firmicutes* phylum (genus *Staphylococcus* and *Streptococcus*), followed by *Actinobacteria*, *Proteobacteria*, and *Bacteroidetes* [26,27,28,29,30].

It is also important to note that the microbiome of the skin, just like the microbiome of other areas, is a dynamic structure that changes, depending on age, gender, environmental factors, and one’s habits, e.g., occupation, use of cosmetics and antibiotics. In normal physiological conditions, the human ecosystem maintains a host–microorganism balance. On the other hand, the interactions between individual microorganisms and those between microorganisms and the host can be a cause of disease. Previous analyses have indicated that the four dominant bacterial phyla living on the skin are: *Actinobacteria*, *Firmicutes*, *Proteobacteria*, and *Bacteroidetes*, with *Corynebacterium*, *Cutibacterium*, and *Staphylococcus* being the most prevalent among over 40 identified bacteria genera [31].

The specific distribution of skin microorganisms by body area may be associated with the characteristic occurrence of individual diseases at individual localizations, e.g., psoriasis typically occurring on the elbows and knees or atopic dermatitis with typical occurrence on skin folds [32]. The skin microbiome shows spatial distribution associated with the skin microenvironment (sebaceous, moist and dry areas) [4]. Thus, in sebaceous areas (face, chest, back), the dominant bacteria are the lipophilic species of the genus *Cutibacterium* and *Staphylococcus*. Bacteria that prefer a humid environment, such as those of the *Staphylococcus* and *Corynebacterium* genera are found in abundance in moist areas (elbow, knee and groin folds), whereas the dry areas of the skin (volar surface of the forearm and the hand) are replete with species belonging to the *Proteobacteria* phylum [31,33].

In addition to bacteria, other microorganisms, such as fungi of the *Malassezia* genus and parasites of the *Demodex* genus, are normally found on human skin. Furthermore, a few studies on the viruses that potentially inhabit the skin indicate that the human virome is also dependent on the skin microenvironment [33,34].

## 4. Microbiome Characteristics in Inflammatory Skin Diseases

The skin microbiome in healthy subjects, as well as the microbiome in inflammatory skin diseases (such as those on the face), has rarely been studied with molecular methods and there is scarce information available. Future research in that area could therefore play an important role in gaining knowledge about the healthy skin microbiome and determining the presence of dysbiosis in patients. However, despite the realization that disturbance of the skin microbiome can lead to a number of pathological conditions and diseases, it is not clear whether skin diseases are the result of a change in the microbiome or whether they cause this change. So far, the skin microbiome has been studied in atopic dermatitis, seborrheic dermatitis, psoriasis, acne, and rosacea (Table 1).

When it comes to the microbiome in other inflammatory skin diseases, there has been a dearth of scientific research [35,36,37,38,39,40]. Understanding the causes and characteristics of skin diseases, along with gathering new insights on the composition of the microbiome, is especially important for physicians in various specialties, such as dermatovenerology, microbiology, infectious diseases, ophthalmology, internal medicine, immunology, and family medicine. This review of the association between changes in the microbiome and skin diseases is intended to contribute to the understanding of disease development and diagnostic and therapeutic procedures in patients, especially in those applying various topical preparations that could alter the skin microbiome. We shall herein present basic information about the microbiome in the most common skin diseases, primarily those that affect the face, with special emphasis on the periorificial region.

## 5. The Skin Microbiome in Patients with Atopic Dermatitis

Atopic dermatitis (AD) is a chronic, recurrent inflammatory skin disease that commonly occurs in children but can also affect adults [41,42,43]. The incidence of AD is increasing, and this disease is thought to affect 5–20% of children and 1–3% of adults worldwide [41,42]. Microbiome studies in patients with AD have shown reduced biodiversity of bacterial communities and a significant increase in colonization by *Staphylococcus aureus*, compared with healthy subjects, in whom colonization by this species is rare. In patients with AD, there is also a difference in the degree of *Staphylococcus aureus* colonization between the lesional and non-lesional areas of the skin, which indicates that the unaffected areas of the skin are predisposed to increased colonization [33,36,44,45,46,47,48,49]. Studies showed that, excluding *Staphylococcus aureus* species, the skin harbors other species of the genus *Staphylococcus*, especially *Staphylococcus epidermidis*. [36,44,50,51]. This species is predominant in patients with milder forms of the disease, while *Staphylococcus aureus* is associated with more severe cases [52]. Patients´ skin showed reduced bacterial biodiversity [41,45,47,49], and the affected skin is poor in bacteria of the genera *Streptococcus, Cutibacterium,* and *Corynebacterium* [36,44]. The microbiota biodiversity microbiome has its role in supporting the rich immune protective milieu of the skin. Coagulase-negative *staphylococci*, such as *Staphylococcus epidermidis*, play a role in immune modulation. Studies have shown that a defect in the ability of effector T cells to produce cytokines, such as IL-17A and IFN-γ, and their reparation are associated with the presence of this species [53,54]. Patients with chronic AD show deficiency in innate defense against *S. aureus*. Structural differences in skin bacterial colonization with coagulase-negative Staphylococci strains, in terms of their reduction, result in reduced antimicrobial peptide production and reduced immune functionality of the skin microbiota [55]. Since antimicrobial peptides LL-37, β-defensins, and dermicidin are present at reduced levels in AD skin, it becomes permissive for *S. aureus* colonization. Additionally, metabolites of adult-associated skin commensals can decrease skin pH and enhance antimicrobial activities, thus suppressing the adherence and growth of *S. aureus* in human keratinocytes [35].

A common site of AD lesions in children is the perioral region. Zheng et al. showed that the affected skin is richest in *Firmicutes* phylum, followed by *Proteobacteria*, *Actinobacteria,* and *Bacteroidetes* phyla [56]. The dominant species on the skin of the perioral region of healthy infants include the bacteria of the genus *Streptococcus* and *Rothia*, whereas in children with AD the abundance of the *Staphylococcus* genus is increased and the *Corynebacterium* genus is reduced. Moreover, the severity of AD positively correlates with decreased *Bateroidetes* and *Fusobacteria* phyla [56]. Further research of AD skin microbiome composition and its functionality will affect treatment options and strategies.

## 6. The Skin Microbiome in Patients with Seborrheic Dermatitis

Seborrheic dermatitis is a chronic inflammatory skin disease that occurs in areas rich in sebaceous glands, most commonly on the scalp and face. The incidence of this disease is considered to be around 3% (although it would be much higher if patients with mild forms of the disease were included) and it is bimodal, occurring in newborns and infants and later during adolescence and adulthood [57]. In HIV positive patients, the incidence reaches up to 85%. [58].

Studies of the skin microbiome in patients with seborrheic dermatitis have shown dysbiosis of the affected skin compared to healthy areas, thereby confirming the previously mentioned changes in the microbiome composition in patients with inflammatory skin diseases. Aside from the bacteria, it is believed that yeasts, especially those of the *Malassezia* genus, play a significant role in the etiopathogenesis of seborrheic dermatitis, through their interactions with the skin, bacteria and the host [40,59,60,61,62,63,64]. However, the mechanisms of these interactions remain unclear. All performed studies have shown that the skin lesions of patients with seborrheic dermatitis are richer in bacteria of the genus *Staphylococcus* [40,59,60,61,63,64,65]. In addition, some studies found that the skin of these patients had a greater abundance in the *Streptococcus*, *Acinetobacter*, and *Pseudomonas* genera, while the genus *Cutibacterium* was less abundant. [40,59,60,61,62,63,64,65]. A study by Park et al. highlighted the increased abundance of the genera *Bacteroides* and *Chryseobacterium* compared to healthy controls [62]. The same applies to the genera *Rhizobium*, *Gordonia*, and *Sphingomonas*.

Saxena at al. performed functional analysis to determine the differences of bacterial and fungal metabolic pathways between healthy and affected scalps and their association with clinical symptoms of seborrheic dermatitis [65].

Bacterial microbiome presented a number of decreased KEGG metabolic pathways, including those related to the metabolism and biosynthesis of vitamins, cofactors, and amino acids and antibiotic resistance, which was in negative correlation with the dandruff score and itching. Commensal bacterial species *Cutibacterium spp*. was shown to carry genes for the synthesis of biotin. Since biotin, vitamin-B6, nicotinate, and lysine demonstrated a negative correlation with dandruff-associated parameters, results of this study highlight the possible beneficial role of bacterial scalp microbiome in supplying essential vitamins and amino acids to the host. Regarding the fungal metabolic pathways, a significant positive correlation of the N-glycan biosynthesis pathway, which is essential for fungal cell wall glycoprotein biosynthesis and adherence to the host surface, was determined with *M. restricta*.

## 7. The Skin Microbiome in Patients with Rosacea

Rosacea is a chronic inflammatory disease characterized by cutaneous and ocular manifestations. The prevalence of rosacea is believed to be around 5%, and it affects people over the age of 30 [66]. Mites of the genus *Demodex* live inside the hair follicles. Two species living on the human skin are *Demodex folliculorum* and *Demodex brevis*. Although they can be found in adults with healthy skin as part of a healthy microbiome, in patients with rosacea there is greater infestation with these mites. They are considered to be a possible trigger of immune response inflammation. The bacterial diversity of the skin affected by rosacea, according to a study by Rainer et al. [67], was higher in comparison to healthy controls, but the difference was not statistically significant. The most abundant species on the skin of patients with rosacea are *Cutibacterium acnes* [67,68,69] and *Staphylococcus epidermidis* [69]. It has been reported that the skin of these patients is richer in certain species of bacteria, such as *Corynebacterium kroppenstedtii*, while the genus *Roseomonas* is reduced [67]. Zaidi et al also did not find a statistically significant change in biodiversity, while a positive and negative correlation was found between the severity of rosacea and abundance of genera *Gordonia* and *Geobacillus* [68]. According to Woo et al., the severity of rosacea increases with age and is associated with a relative decrease in the abundance *of Cutibacterium acnes* and an increase in the prevalence of *Snodgrassella alvi* [69]. Antibiotic treatment reduces the severity of the disease and increases the abundance of *Weissell confus*. The results of studies on bacterial microbiota in other inflammatory skin diseases have stimulated microbiome research in patients with rosacea, but currently only a few studies have been reported, without significant results. Further research is needed.

## 8. The Skin Microbiome in Patients with Acne

Acne is a very common inflammatory skin disease, with an estimated prevalence of 30–95% in adolescents. The findings of previous cultivation-based studies indicated that acne disease is the result of the proliferation of *Cutibacterium acnes*. However, recent studies have shown that there is no difference in the abundance of *Cutibacterium acnes* between acne-prone skin and the seborrheic sites of the skin of healthy controls, and that acne-prone skin has a reduced number of different *Cutibacterium acnes* phylotypes [70,71,72,73,74]. According to a study by Barnard et al., the abundance of the genus *Cutibacterium-Cutibacterium acnes* and *Cutibacterium granulosum* is even slightly higher on healthy skin compared to acne-affected skin [73]. The skin of patients with acne (i.e., on the surfaces of comedones, papules and pustules) predominantly harbors bacteria of the genera *Firmicutes* and *Staphylococcus*, though mostly *Staphylococcus epidermidis* [72,75] and phylum *Proteobacteria* [72], while the presence of *Actinobacteria* phlyum is reduced [72,73]. Additionally, due to metagenomic shotgun analysis, this study established an important concept of disrupted balance in the metagenomic elements of the microbiota, influencing disease pathogenesis. Among identified operational gene units (OGUs), functional profiles differed between acne patients and healthy individuals. In acne patients, genetic elements involved in cell viability, virulence, and immunity, such as genetic units coding thiopeptide bacteriocin (family of microcins antimicrobial peptides) precursor synthesis and transport, were significantly more abundant. Other genetic elements, like pathogenicity islands previously associated with acne, and locus involved in recombination and chromosome transformation with cluster of streptolysin S-associated genes (sag) involved in the biosynthesis and transport of a bacterial toxin were highly abundant in patients with acne.

In contrast to the enrichment of virulence-related genes observed in the acne metagenome, genes involved in microbial metabolism and nutrient biosynthesis were significantly more abundant in the healthy metagenome [73].

According to a study by Kelhala et al., the genera *Streptococcus*, *Gamella*, *Fusobacterium*, *Granulicatella*, and *Neisseria* are reduced on the skin of patients with acne, probably due to the relative overgrowth of bacteria of the genus *Cutibacterium*, which limits the growth of other bacteria by competing for the same ecological niche [71]. The genus *Cutibacterium* makes up less than 2% of the bacteria on acne lesions [72]. It has been reported that the amount of *Staphylococcus* genus is in positive correlation with the severity of the disease. Based on these findings, many studies have attempted to identify the *Cutibacteruim acnes* phylotypes associated with this disease. The acne-related phylotype IA1 increases the pathogenic effect of these bacteria due to the it’s inflammatory potential, differences in virulence generation and biofilm production [74,76,77]. Recent studies have shown that the most severe stages of the disease are associated with an increase in bacteria belonging to the *Faecalibacterioma*, *Klebsiella*, *Odirobacter,* and *Bacteroides* genera [78]. In conclusion, according to present data, acne pathogenesis can be related to balance and its disruption in the healthy and acne-affected skin microbiome, including bacterial species and metagenomic elements. 

## 9. Perioral/Periorificial Dermatitis

Perioral/periorificial dermatitis is characterized by small, inflamed papules that occur on the skin around the mouth, which can also affect the area around the eyes and nose. Some authors prefer the term “periorificial dermatitis”, which includes both perioral and periocular dermatitis, since the term perioral refers specifically to the area around the mouth and is therefore less appropriate. The etiopathogenesis of this disease is not sufficiently clear; it seems to be associated with the use of local corticosteroids and irritants, such as makeup, moisturizers, sunscreens, tonics etc. In addition to the afore mentioned extrinsic factors, a possible cause might be the epidermal barrier disorder and an atopy tendency [79,80,81]. Periorificial dermatitis most commonly affects women between the ages of 16 and 45, but it can also occur in the elderly, among men and children from seven months to 13 years of age [82]. A special subtype of periorificial dermatitis known as granulomatous periocular dermatitis affects children only.

Aside from the perioral region, the lips are often affected by inflammation (cheilitis). Cheilitis is an inflammatory condition of the lips that can occur in many inflammatory skin diseases, as well as in systemic diseases, such as lupus erythematosus, lichen planus, AD, etc. It can also develop as an isolated condition or as part of conditions such as anemia (vitamin B12 and iron deficiency) or local infections (e.g., herpes and oral candidiasis), due to contact reaction to an irritant or allergen, or it can be triggered by exposure to the sun (actinic cheilitis) or medications (especially retinoids) [83].

According to data gathered on the epidermal barrier disorder observed in patients with perioral dermatitis, microbiome research could provide important conclusions about the microbiome of the perioral region. To date, studies on the skin microbiome of the periorificial region are few. Zheng et al. found that bacteria of the genera *Streptococcus* and *Rothia* predominate on the skin of the perioral region of healthy infants [56].

## 10. Periocular Dermatitis

Periocular dermatitis (PD) refers to skin changes in the periocular region, which are relatively common in clinical practice, as a number of different diseases can occur in this area, often with limited diagnostics that rarely take the microbiological composition of the skin into account. These skin changes occur most often in AD, seborrheic dermatitis, contact dermatitis, rosacea, and psoriasis [84,85], as well as certain infectious diseases such as erysipelas, impetigo, lues, zoster, HIV and other localized and systemic bacterial diseases, viral infections, and fungal infections [86]. According to recent studies, the largest number of patients who present with PD have contact dermatitis, followed by AD and irritative dermatitis, and less often psoriasis, seborrheic dermatitis, rosacea, and dermatomyositis. [87,88,89,90,91].

Sometimes it is difficult to distinguish the etiology of these skin changes, as well as the role of the microbiome in their development. Given the lack of clear diagnostic indicators in clinical practice that would confirm an underlying specific skin disease, the condition is usually characterized descriptively, as periocular dermatitis. The risk factors for PD development include female gender, over 40 years of age, and a tendency toward atopy [86]. The onset of PD is associated with several factors, such as epidermal barrier disorder, innate immune system activation and changes in the skin microbiome. Pathogenetic activities in the skin of the eyelids are particularly important for the microbiome of the periocular region. It contains glands (Meibomian, Zeiss, and Moll) that prevent the evaporation of the tear film, produce secretory components and are a vital component of the immune defense against pathogenic microorganisms [92]. The microbiome of healthy periocular skin harbors coagulase negative *Staphylococci* (*Staphylococcus epidermidis), Staphylococcus aureus,* and *Cutibacterium acnes*, whose presence is not always considered pathological but may play a role in Meibomian gland dysfunction [93,94]. Another common finding on the skin of the periocular region is *Demodex* mite which is observed in healthy individuals but even more common in patients with blepharitis, where it plays a yet insufficiently elucidated role [94]. The skin of the periocular region in healthy individuals is inhabited mostly by bacteria from the phyla *Actinobacteria*, followed by *Firmicutes*, *Proteobacteria*, and *Bacteroidetes*, which corresponds to the findings of other seborrheic skin localizations [31,95].

## 11. Skin Microbiome in Patients with Psoriasis

Psoriasis is an immune-mediated chronic inflammatory skin disease, affecting 0.5% to 11.4% of adults and 0% to 1.37% of children worldwide [96]. The results of skin microbiome studies in patients with psoriasis are not fully consistent [89,90,91,92,93,94,95]. Studies by Alekseyenko et al., Wang et al., and Langan et al. showed that the biodiversity in psoriatic lesions is reduced compared to healthy skin [97,98,99]. A study by Chang et al. found increased biodiversity in skin affected by psoriasis, while a study by Fahlen et al. found no difference [100,101]. The most abundant bacteria harboring psoriatic lesions are the bacteria of the *Firmicutes* phylum which are present on psoriatic skin in a larger proportion than on the skin of healthy subjects [101,102], whereas the phyla *Actinobacteria* [98,99,100,101,102] and *Proteobacteria* are reduced [102]. Aside from that, studies show an increase in the abundance of *Streptococcus* [102] and *Staphylococcus* genera [6,99], i.e., certain species of *Staphylococcus aureus*, *Staphylococcus pettenkoferi,* and *Staphylococcus sciuri*, and the depletion of the genus *Cutibacterium*, *Staphylococcus epidermidis*, *Cutibacterium acnes,* and *Cutibacterium granulosum* species [100,102].

Alekseyenko et al. found that the genera *Corynebaterium*, *Cutibacterium*, *Staphylococcus,* and *Streptococcus* are more abundant in patients with psoriasis, while the genera *Cupriavidus*, *Methylobacterium* and *Schlegelella* are less abundant [97]. It has also been shown that there are two types of psoriasis, based on the abundance of certain bacteria, i.e., type 1, with the predominance of *Proteobacteria* phylum and type 2, with the predominance of *Firmicutes* and *Actinobacteria* phyla. Fahlén et al. analyzed the microbiome using skin bioptates and showed that the phylum *Proteobacteria* was more prevalent on the trunks of patients with psoriasis than on those of healthy subjects, while the genera *Cutibacterium* and *Staphylococcus* were reduced on the affected skin of the limbs [101]. The results of their study could be controversial due to different sampling techniques used in microbiome analysis, i.e., skin biopsy in the study by Fahlen et al vs. skin swab in the above mentioned studies. The results of studies performed by Wang et al. showed that the bacterial families *Campylobacteraceae* and *Gordoniaceae* are associated with a change of skin status from healthy to psoriatic [98]. 

Using metagenomic analysis in exploring bacterial diversity associated with psori-atic lesions, Tett et al. observed several significant functional microbiome differences in relation to disease. Biological (KEGG) pathways involved primarily in biodegradation and metabolism were increased in psoriatic lesions compared to unaffected skin, including benzoate, naphthalene, and lysine degradation. In unaffected skin areas, genetic elements related to the metabolism of vitamins, cofactors, and lipid metabolism were more prevalent. Such differences in broad functionality likely reflect nutrient availability on dis-ease-affected and unaffected skin. Additionally, they identified that bacterial secretion and protein export are more prevalent in unaffected skin microbiomes, compared to those affected by disease [6]. All these findings concerning psoriasis-related microbiome composition and mapping present the potential for the discovery of new disease biomarkers and therapeutic options.

## 12. Conclusions

Diagnosing a specific skin disease in different areas of the skin is a great challenge in clinical practice and the therapeutic approach is often undefined. According to recent research using molecular methods, the diversity of the skin microbiota is even greater than has previously been recognized by conventional cultivation methods, which have led to underestimation of the diversity of certain groups of bacteria. As manifestations of inflammatory skin diseases on the face are frequent, understanding the causes and development of such diseases is particularly important for physicians in various specialties, such as dermatovenerology, microbiology, infectious diseases, ophthalmology, and internal medicine. Once the changes in the structure and diversity of the skin microbiota associated with individual diseases are identified, their detection for diagnostic purposes could be performed by simpler and cheaper molecular methods. Thus, research on possible changes in the microbiome would contribute to a better understanding of the development of the disease, diagnostics, and therapeutics, especially in patients using various topical preparations that could alter the skin microbiome. Identifying the bacterial markers associated with a particular inflammatory skin diseases would significantly accelerate the diagnostic procedure and reduce the cost of treatment. Microbiota research and determination could lead to the identification of potential causes of skin diseases that have passed unnoticed by simpler methods, which would help in the design and development of a more effective treatment. 

Our review is designed to be an overview of current data and knowledge that would serve as basis for future research. Specific imbalances in microbiota composition in patients with inflammatory skin diseases exist, but the results of studies performed on the matter are inconsistent, and there is a possibility that the changes in the microbiome are a consequence, and not the cause of the disease. Given the limited research performed on skin microbiome due to challenges that are still present concerning the optimization and standardization of sampling and isolation procedures, as well as data evaluation and bioinformatic analysis, we believe that an overview of all that is known and unknown to the scientific community at this point regarding the skin microbiome could help in future research design.

In order for the accumulated knowledge on the microbiome to be applied in diagnostics, and especially in therapy, it is necessary to first determine which microorganisms may be beneficial. It used to be thought that there was a division according to “bad” and “good” bacteria, but it is now clear to us that particular strains can have protective effects on skin health, whereas others can potentially contribute to disease development. Obtaining data/knowledge on microbiome composition can therefore be used for therapeutic actions on the skin disease-specific microbiome, e.g., probiotics, systemic drugs, and topical medication that act on the microbiome specifically for a particular skin disease. Prebiotics and probiotics have long been used in the modulation of the intestinal microbiome, while the use of topical probiotics for the treatment of AD and acne is currently in the phase of efficacy testing in humans. Probiotics are also already in use in fighting against skin aging, and such products are commercially available in the form of creams and serums. Further research on the skin microbiome in inflammatory skin diseases is needed, particularly concerning the impact of antibiotics on the microbiome and the beneficial effects of topical probiotics in improving skin health and the individualized treatment of skin diseases.

## Figures and Tables

**Table 1:** Microbiome shifts in most common inflammatory skin diseases.

Atopic dermatitis	↑ *Staphylococcus spp.* ^1 3 7 8^ ↑ *Staphylococcus aureus* ^1 2 3 4 5 6 8^ ↑ *Staphylococcus epidermidis* ^1 4 7^	↓ *Streptococcus spp.* ^1^ ↓ *Cutibacterium spp.* ^1 3^ ↓ *Corynebacterium spp.* ^1 3^
Psoriasis	↑ *Firmicutes* ^9 10^ ↑ *Proteobacteria* ^11 14^ ↑ *Streptococcus spp.* ^9^ ↑ *Prevotella* ^10^ ↑ *Staphylococcus spp.* ^10 13^ ↑ *Staphylococcus aureus* ^11^ ↑ *Staphylococcus pettenkoferi* ^11^ ↑ *Staphylococcus sciuri* ^11^	↓ *Actinobacteria* ^9 10 11 12^ ↓ *Gordoniaceae* ^11^ ↓ *Proteobacteria* ^9^ ↓ *Staphylococcus epidermidis* ^11^ ↓ *Cutibacterium spp.* ^9 10 14^ ↓ *Staphylococcus spp.* ^14^ ↓ *Cutibacterium acnes* ^11^ ↓ *Cutibacterium granulosum* ^11^
Seborrheic dermatitis	↑ *Staphylococcus spp.* ^15 16 17 18 19 20 21 22^ ↑ *Staphylococcus epidermidis* ^20^ ↑ *Streptococcus spp.* ^18^ ↑ *Pseudomonas spp.* ^22^ ↑ *Acinetobacter* ^18^	↓ *Cutibacterium spp.* ^15 16 17 19 20 21^
Acne	↑ *Firmicutes ^24 25^* ↑ *Proteobacteria* ^23 24^ ↑ *Staphylococcus spp.* ^24 25^	↓ *Actinobacteria ^23 24^* ↓ *Cutibacterium spp. ^23^* ↓ *Cutibacterium acnes* ^23^ ↓ *Cutibacterium granulosum ^23^*
Rosacea	↑ *Corynebacterium kropp* ^26^ ↑ *Gordonia* ^27^ ↑ *Geobacillus* ^27^	↓ *Rosemonas spp.* ^26^

1 Kong et al, 2012, 2 Gonzalez et al, 2016, 3 Shi et al, 2016, 4 Clausen et al, 2017, 5 Baurecht et al, 2018, 6 Callewaert et al, 2020, 7 Seite et al, 2014, 8 Kim et al, 2017, 9 Gao et al, 2008,10 Langan et al, 2019, 11 Chang et al, 2018, 12 Wang et al, 2020, 13 Tett et al, 2017, 14 Fahlén et al 2012, 15 Clavaud et al, 2013, 16 Wang et al, 2015, 17 Xu et al, 2016, 18 Tanaka et al, 2016, 19 Park et al, 2017, 20 Saxena et al, 2018, 21 Grimshaw et al, 2019, 22 Lin et al, 2021, 23 Barnard et al, 2016, 24 Dreno et al, 2017, 25 Kim et al, 2021, 26 Rainer et al, 2020, 27 Zaidi et al, 2018. ↑ higher abundance in lesional than in non-lesional skin; ↓ lower abundance in lesional than in non-lesional skin.
